# Mixture of MMP-2, MLC, and NOS Inhibitors Affects NO Metabolism and Protects Heart from Cardiac I/R Injury

**DOI:** 10.1155/2020/1561478

**Published:** 2020-04-07

**Authors:** Anna Krzywonos-Zawadzka, Aleksandra Franczak, Grzegorz Sawicki, Iwona Bil-Lula

**Affiliations:** ^1^Department of Medical Laboratory Diagnostics, Division of Clinical Chemistry and Laboratory Hematology, Wroclaw Medical University, Wroclaw, Poland; ^2^Department of Anatomy, Physiology and Pharmacology, College of Medicine, University of Saskatchewan, Saskatoon, Canada

## Abstract

**Objectives:**

Coronary reperfusion procedure leads to ischemia/reperfusion injury of the heart (IRI). IRI arises from increased degradation of myosin light chains and increased activity of matrix metalloproteinase 2 (MMP-2). Increased production of toxic peroxynitrite (ONOO^−^) during oxidative stress is a source of increased nitration/nitrosylation of contractile proteins, which enhance their degradation through MMP-2. Hence, an imbalance in nitric oxide (NO) metabolism along with oxidative stress is an important factor contributing to pathophysiology of cardiovascular disorders, including myocardial infarction. The aim of the current study was to provide an important insight into understanding the interaction of iNOS, eNOS, and ADMA during oxidative stress and to propose the beneficial therapy to modulate this interaction. *Material and Methods*. Pathogen-free Wistar rats were used in this study as a surrogate heart model *ex vivo*. Rat hearts perfused using the Langendorff method were subjected to global no-flow ischemia with or without administration of DOXY (1 *µ*M), ML-7 (0.5 *µ*M), and L-NAME (2 *µ*M) mixture. Haemodynamic parameters of heart function, markers of I/R injury, tissue expression of iNOS, eNOS, and phospho-eNOS, asymmetric dimethylarginine, and NO production as well as MMP-2 activity were measured.

**Results:**

Mechanical heart function and coronary flow (CF) were decreased in the hearts subjected to I/R. Treatment of the hearts with the tested mixture resulted in a recovery of mechanical function due to decreased activity of MMP‐2. An infusion of Doxy, ML-7, and L-NAME mixture into I/R hearts decreased the expression of iNOS, eNOS, and phospho-eNOS and in consequence reduced ADMA expression. Decreased ADMA production led to enhanced NO synthesis and improvement of cardiac function at 85% of aerobic control.

**Conclusions:**

Synergistic effect of the multidrug therapy with the subthreshold doses allows addressing a few pathways of I/R injury simultaneously to achieve protection of cardiac function during I/R.

## 1. Introduction

Coronary reperfusion is a standard procedure for the treatment of patients with myocardial infarction [1]. However, subsequently, it leads to ischemia/reperfusion injury (IRI), resulting in a cascade of processes detrimental to cardiac tissue. Impaired nitric oxide (NO) metabolism along with oxidative stress is known to be an important factor contributing to the pathophysiology of cardiovascular disorders, including myocardial infarction [2–5].

NO is synthesized from L-arginine through a complex oxidation reaction catalyzed by NO synthase (NOS) and plays an important role in cardiovascular homeostasis. The levels and bioactivity of NO are regulated by eNOS (endothelial NOS), nNOS (neuronal NOS), and iNOS (inducible NOS) as well as endogenous NOS inhibitors such as asymmetric dimethylarginine (ADMA) [6]. Physiological amounts of NO and its metabolites (nitrites) mediate cardioprotection [6]. However, oxidative stress during IRI triggers the increased expression of iNOS and subsequent production of very potent ONOO^−^ (peroxynitrite) which promote cell damage [7]. CVD (cardiovascular disease) is associated not only with increased iNOS but also with an increase in eNOS expression [6]. Moreover, it was shown that the ADMA level was increased in many CVDs—myocardial infarction, among others [8, 9]. ADMA was shown to cause NOS uncoupling which is associated with the production of superoxide anion (O_2_^•−^) instead of NO [10]. There is evidence that the L-arginine/NO/ADMA pathway plays a role in the development of CVD [3, 4, 8].

It is also well known that ROS (such as ONOO^−^) is generated during IRI activate matrix metalloproteinase-2 (MMP-2) [11]. MMP-2 degrades contractile proteins including myosin light chains (MLCs), resulting in contractile dysfunction [12]. Furthermore, posttranslational modifications (nitration/nitrosylation by ONOO^−^ and phosphorylation triggered by IRI) of MLCs enhance their degradation by MMP-2 [12–14].

The current preclinical studies focus on multidrug therapies in the low concentrations of drugs in order to inhibit pathological processes, while avoiding the alterations of physiological roles of targeted proteins [15]. The aim of this study was to verify if coadministration of subthreshold doses of doxycycline (MMP-2 inhibitor), L-NAME (nonselective inhibitor of NOS), and ML-7 (inhibitor of MLC phosphorylation by MLCK, myosin light chain kinase) regulates NOS-ADMA-NO pathway leads to cardioprotection.

## 2. Materials and Methods

This investigation conforms to the Guide to the Care and Use of Experimental Animals published by the Polish Ministry of Science and Higher Education. This study was approved by the Ethics Committee for Experiments on Animals at the Ludwik Hirszfeld Institute of Immunology and Experimental Therapy Polish Academy of Sciences, Wroclaw, Poland, no. 115/2017/P1.

### 2.1. Experimental Animals

Pathogen-free Wistar rats (supplied by the Mossakowski Medical Research Center, Polish Academy of Sciences, Warsaw, Poland) weighing 300–350 g were used in this study. The animals were kept in cages (2 rats/cage) under a light/dark (12/12) cycle at controlled temperature (22 ± 2°C) and humidity (55 ± 5%) and were allowed *ad libitum* access to food and water.

### 2.2. Pharmacological Agents

N(G)-nitro-L-arginine methyl ester (L-NAME), doxycycline (Doxy), and 1-(5-Iodonaphthalene-1-sulfonyl)-1H-hexahydro-1,4-diazepine hydrochloride (ML-7) (Sigma-Aldrich, Saint Louis, Missouri, USA) were dissolved in ethanol/ddH_2_O, and then, immediately before administration, diluted with Krebs–Henseleit buffer to a final concentration. The ethanol concentration infused into the heart was equal to 0.025% (*v/v*), and it was experimentally confirmed that such concentration does not affect the mechanical function of the heart. Final concentrations of drugs were chosen based on previous experiments [16].

### 2.3. Langendorff Isolated Heart Perfusion

**Figure 1:**
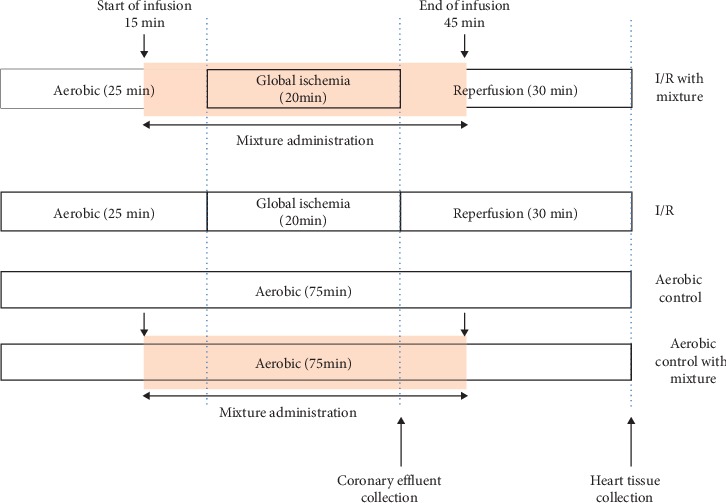
Experimental protocol for ischemia/reperfusion (I/R) and aerobic control with or without administration of the mixture of doxycycline (MMP-2 inhibitor; 1.0 *µ*M), L-NAME (NOS inhibitor; 2 *µ*M), and ML-7 (inhibitor of MLC phosphorylation; 0.5 *µ*M).

The hearts were excised from animals desensitized with buprenorfin (0.05 mg/kg, i.p.) and anaesthetized with sodium pentobarbital (40 mg/kg, i.p.). Spontaneously beating isolated hearts were rinsed by immersing in the ice-cold Krebs–Henseleit buffer (118 mmol/l NaCl, 4.7 mmol/l KCl, 1.2 mmol/l KH_2_PO_4_, 1.2 mmol/l MgSO_4_, 3.0 mmol/l CaCl_2_, 25 mmol/l NaHCO_3_, 11 mmol/l glucose, and 0.5 mmol/l EDTA, and pH 7.4), and immediately after removal they were cannulated by the aorta on a Langendorff apparatus and maintained at 37°C. The hearts were perfused in the Langendorff system at a constant pressure of 60 mmHg with Krebs–Henseleit buffer and gassed continuously with a mixture of 95% O_2_ and 5% CO_2_. After stabilization (25 min), the hearts were subjected to global, no-flow ischemia (20 min) and then aerobic reperfusion (30 min) (Figure 1). Mixture of drugs was administered 10 min before occlusion and first 10 min during reperfusion. Coronary flow (CF), heart rate (HR), and left ventricular developed pressure (LVDP, systolic minus diastolic ventricular pressures) were monitored and registered. Cardiac mechanical function was expressed as the product of HR and LVDP—rate pressure product (RPP) at 75 min versus 25 min of perfusion. The isolated hearts were immediately submerged in liquid nitrogen and stored in −80°C for further analysis.

### 2.4. Preparation of Heart Tissue Homogenates

Previously frozen hearts were crushed in liquid nitrogen and homogenized by mechanical homogenization in ice-cold buffer (50 nM Tris-HCl (pH 7.4), 3.1 mM sucrose, 1 nM dithiothreitol, 10 *µ*g/ml leupeptin, 10 *µ*g/ml soybean trypsin inhibitor, 2 *µ*g/ml aprotinin, and 0.1% Triton X-100). The homogenates were centrifuged at 30 000xg at 4°C for 10 min, and the supernatants were collected and stored at −80°C for further analysis. Total protein concentration in cardiac tissue homogenates was determined using the Bradford method (Bio-Rad), and bovine serum albumin (heat shock fraction, 98%; Sigma-Aldrich) was used as the protein standard.

### 2.5. Measurement of MMP-2 Activity

Gelatin zymography was performed with the protocol of Heussen and Dowdle modified by us [17]. Briefly, samples containing 20 *µ*g of protein were mixed with 4x Laemmli sample buffer (Bio-Rad, Hercules, California, USA) and applied to 8% polyacrylamide gel copolymerized with 2 mg/ml gelatin and 0.1% SDS. After electrophoresis, gels were rinsed in 2.5% Triton X-100 (three times for 20 min). Then, gels were washed in incubation buffer (50 mM Tris-HCl, 5 mM CaCl_2_, 150 mM NaCl, and 0.05% NaN_3_) for 20 min at room temperature and incubated overnight in incubation buffer at 37°C. Gels were stained in staining solution (0.05% Coomassie Brilliant Blue G-250, 50% methanol, and 10% acetic acid) and destained in destaining solution (30% methanol and 10% acetic acid v: v). Zymograms were scanned using VersaDoc 5000 (Bio-Rad), and the band intensities were analyzed by Quantity One software *v*. 4.6.6 (Bio-Rad). MMP activity was expressed in arbitrary units (AU) as activity per microgram of total protein.

### 2.6. Measurement of LDH Activity

Concentration of LDH (lactate dehydrogenase; marker of cell damage, released into extracellular space as a result of membrane damage/permeability) in coronary effluents was measured using Lactate Dehydrogenase Activity Assay Kit (Sigma-Aldrich) according to manufacturer's instruction. Briefly, LDH catalyses the interconversion of pyruvate and lactate with the reduction of NAD to NADH, which is detected with a colorimetric reaction at 450 nm.

### 2.7. Measurement of iNOS, eNOS, and Phosphorylated eNOS

The tissue expression of iNOS, eNOS, and phosphorylated eNOS (phospho S1177) was determined using western blot. The aliquots of 60 *µ*g of total protein from heart homogenates were separated on 12% SDS-PAGE. Then, iNOS, eNOS, and phospho-eNOS proteins were transferred on PVDF membranes (Bio-Rad) and were detected with primary antibody (mouse anti-iNOS polyclonal antibody 1 : 5000 (Abcam, ab21775); anti-eNOS (Abcam, ab50010); anti-phospho (S1177)-eNOS (Abcam, ab75639), respectively) and secondary goat anti-mouse IgG horseradish peroxidase conjugate 1 : 1000 (Bio-Rad, STAR207P). Clarity^TM^ Western ECL (Bio-Rad) substrate was used for proteins detection. ChemiDoc^TM^ MP System and Quantity One Software (Bio-Rad) were used for detection of bands and measurement of their density. iNOS, eNOS, and phospho-eNOS quantities were expressed as AU normalized to total protein amount.

### 2.8. Measurement of Nitric Oxide (NO)

The concentration of NO in cardiac tissue was assessed by Nitric Oxide Assay Kit (Abcam, ab65327), which measures the amount of total nitrate/nitrite in a two-step reaction. Firstly, nitrates were converted to nitrites by nitrate reductase, and secondly, nitrites were converted into a colored azo compound detected spectrophotometrically at 540 nm. The NO content in cardiac tissue was expressed as nmol per mg of total protein. The detection limit of this assay was 0.03 uM.

### 2.9. Measurement of Endogenous Asymmetrical Dimethylarginine (ADMA)

The content of ADMA in cardiac tissue was determined by using the Rat ADMA ELISA kit (Cusabio, Houston, USA). Briefly, it was a competitive assay, in which the plate was precoated with goat-anti rabbit antibody which did bind ADMA from cardiac tissue or HRP-conjugated ADMA. The intensity of the color developed after adding a substrate solution was opposite to the amount of ADMA in the sample. ADMA content in cardiac tissue was expressed as ng per mg of total protein. The minimum detectable dose was less than 0.75 ng/ml.

### 2.10. Statistical Analysis

The independent samples *t*-test was performed. ANOVA with Tukey's as a post hoc test was used for multiple comparisons, as appropriate. The Shapiro–Wilk test was used to test the assumption of normality. Correlations were assessed using Pearson's test. Data are presented as mean ± SEM. A *p* value less than 0.05 was used as a level of statistical significance. The statistical analysis was performed using GraphPad Prism *v*.8.

## 3. Results

### 3.1. Effect of Coadministration of Subthreshold Doses of Inhibitors of MMP-2, MLCK, and NOS on Cardiac Mechanical Function

**Figure 2:**
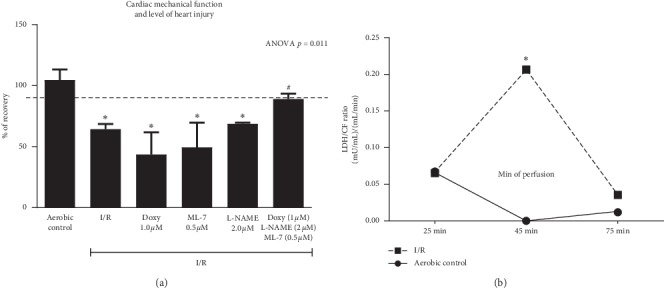
An effect of coadministration of doxycycline (MMP-2 inhibitor; 1.0 *µ*M), L-NAME (NOS inhibitor; 2 *µ*M), and ML-7 (inhibitor of MLC phosphorylation; 0.5 *µ*M) on recovery of mechanical function of I/R hearts (a). An effect of I/R on the LDH level in coronary effluents (b). Doxy, doxycycline; MMP-2, matrix metalloproteinase-2; NOS, nitric oxide synthase; MLC, myosin light chain; I/R, ischemia/reperfusion; LDH, lactate dehydrogenase; ^*∗*^*p* < 0.05 vs. aerobic control; #*p* < 0.05 vs. I/R; mean ± SEM.

Cardiac mechanical function was significantly decreased (by approximately 40%) in the hearts subjected to I/R in comparison to the hearts perfused aerobically (Figure 2(a)). The heart failure was the result of substantial heart injury during I/R measured by LDH content in coronary effluents (Figure 2(b)). Mixture of Doxy (1.0 *µ*M), ML-7 (0.5 *µ*M), and L-NAME (2 *µ*M) increased heart function at 85% of aerobic control. The cardioprotective effect was not observed when drugs were administered separately (Figure 2(a)).

### 3.2. An Influence of Inhibitors Mixture on NOS/ADMA/NO Pathway

**Figure 3:**
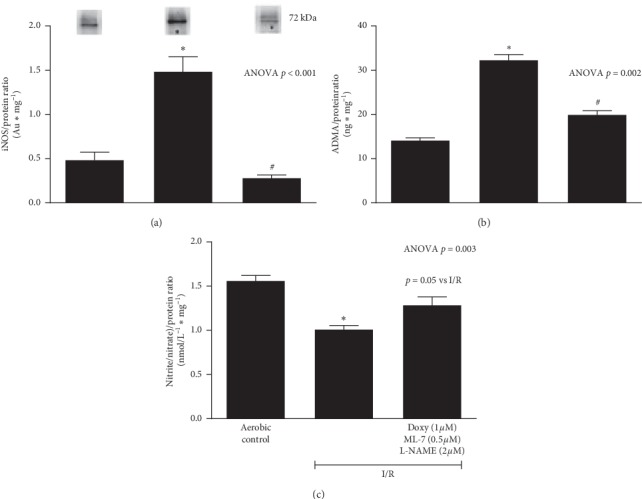
An effect of coadministration of doxycycline (1.0 *µ*M), L-NAME (2 *µ*M), and ML-7 (0.5 *µ*M) on expression of iNOS (a), ADMA (b) and NO production (as total nitrite/nitrate) (c) in cardiac tissue. iNOS, inducible nitric oxide synthase; ADMA, asymmetric dimethylarginine; NO, nitric oxide; I/R, ischemia/reperfusion; ^*∗*^*p* < 0.05 vs. aerobic control; #*p* < 0.05 vs. I/R; mean ± SEM.

**Figure 4:**
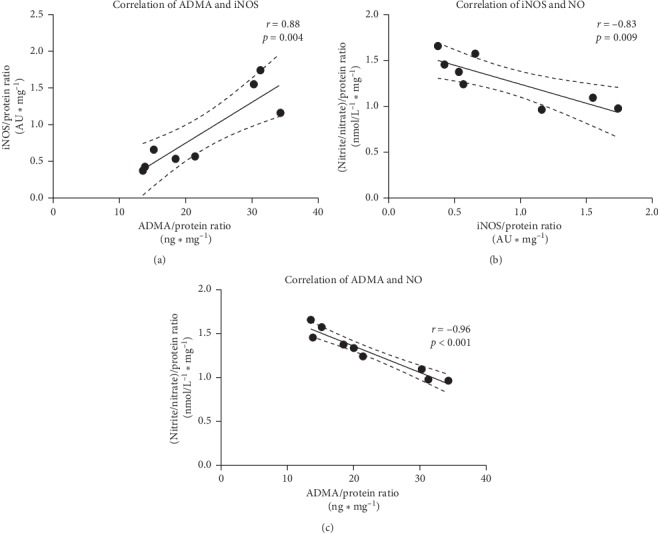
Correlations between iNOS, ADMA, and NO (a–c). iNOS, inducible nitric oxide synthase; ADMA, asymmetric dimethylarginine; NO, nitric oxide (measured indirectly as nitrite/nitrate).

**Figure 5:**
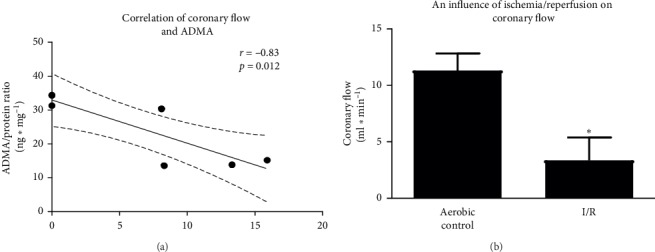
Correlation between coronary flow and ADMA (a). An effect of I/R on coronary flow (b). ADMA, asymmetric dimethylarginine; I/R, ischemia/reperfusion; ^*∗*^*p* < 0.05 vs. aerobic control.

**Figure 6:**
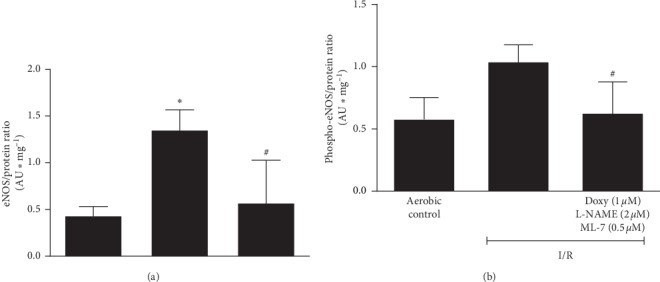
An effect of coadministration of doxycycline (1.0 *µ*M), L-NAME (2 *µ*M), and ML-7 (0.5 *µ*M) on expression of eNOS (a) and phospho-eNOS (b) in cardiac tissue. eNOS, endothelial nitric oxide synthase; phospho-eNOS, phosphorylated (S1177) eNOS; I/R, ischemia/reperfusion; ^*∗*^*p* < 0.05 vs. aerobic control; ^#^*p* < 0.05 vs. I/R; mean ± SEM.

The significantly increased levels of iNOS (Figure 3(a)) and ADMA (Figure 3(b)) were observed in the hearts subjected to I/R compared to aerobic controls. Conversely, I/R led to decrease of NO level (measured indirectly by total nitrite/nitrate content) (Figure 3(c)). The coadministration of subthreshold doses of inhibitors led to reduction of iNOS and ADMA levels to the level approximate to aerobic control (Figures 3(a)–3(b)) and in turn increase in NO content to the level close to the aerobic control (Figure 3(c)):

- The positive correlation between iNOS and ADMA was found (Figure 4(a)). Level of both iNOS and ADMA negatively correlated with NO content (Figures 4(b) and 4(c), respectively).
- The analysis of correlations showed that ADMA was negatively correlated with CF (Figure 5(a)). CF was found significantly lower in I/R compared to aerobic controls (Figure 5(b)).
- Moreover, in the hearts subjected to I/R, increased levels of eNOS (Figure 6(a)) and phospho-eNOS (Figure 6(b)) were observed. After coadministration of inhibitors, the levels were significantly reduced to the levels approximate to aerobic control (Figures 6(a)–6(b)).

### 3.3. Effect of Coadministration of Subthreshold Doses of Inhibitors of MMP-2, MLCK, and NOS on MMP-2 Activity

**Figure 7:**
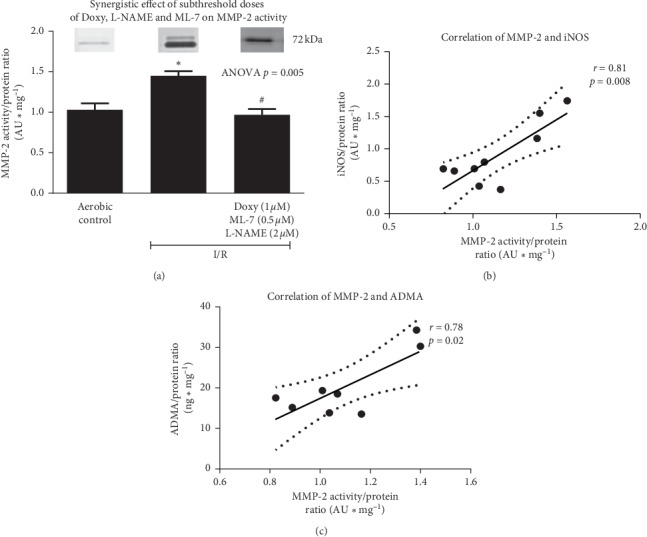
An effect of coadministration of doxycycline (1.0 *µ*M), L-NAME (2 *µ*M), and ML-7 (0.5 *µ*M) on activity of MMP-2 in cardiac tissue (a). Correlation between MMP-2 and iNOS (b) and MMP-2 and ADMA (c). MMP-2, matrix metalloproteinase-2; iNOS, inducible nitric oxide synthase; ADMA, asymmetric dimethylarginine; I/R, ischemia/reperfusion; ^*∗*^*p* < 0.05 vs. aerobic control; ^#^*p* < 0.05 vs. I/R; mean ± SEM.

The activity of MMP-2 in cardiac tissue of rats subjected to I/R was significantly higher compared to aerobic controls. Coadministration of subthreshold doses of Doxy (1.0 *µ*M), ML-7 (0.5 *µ*M), and L-NAME (2 *µ*M) led to normalization of MMP-2 activity to the level of aerobic control (Figure 7(a)). There was a positive correlation between MMP-2 and iNOS (Figure 7(b)) as well as MMP-2 and ADMA (Figure 7(c)).

## 4. Discussion

The pathophysiology of ischemia/reperfusion injury is very complex, and thus, it requires multisited actions to achieve desired therapeutic effects [15]. The main contributors to IRI are increased oxidative stress [11] and subsequent increased expression of NOS [18], activation of MMPs [19], and enhanced post-translational modifications of contractile proteins, which make them more susceptible to proteolytic degradation [20]. In order to target the main molecular pathway of IRI, in this study we simultaneously administered the subthreshold doses of the following drugs: doxycycline (MMP-2 inhibitor; 1.0 *µ*M), L-NAME (NOS inhibitor; 2 *µ*M), and ML-7 (inhibitor of MLC phosphorylation; 0.5 *µ*M).

The role of the NOS/ADMA/NO pathway in myocardial IRI is multifarious and fairly perplexing [21]. NO is an important molecule in physiological conditions due to its antioxidant, vasodilator, anti-inflammatory, and antiplatelets effects [22, 23]. Moreover, NO may serve cardioprotective in ischemia-induced late preconditioning [24]. However, there is growing evidence of detrimental role of activation of the NOS/ADMA/NO pathway during ischemia/reperfusion [21]. The potential regulation of the NOS/ADMA/NO pathway during oxidative stress has been previously described [25]. The adverse consequences are likely due to imbalance between NO and ONOO^−^. The latter one comes from the reaction of NO with superoxide (O_2_^•−^) [7]. Peroxynitrite causes nitration/nitrosylation of myocardial proteins [26] and activates MMP-2 [11], leading in turn to enhanced degradation of proteins and contractile dysfunction [19].

In this study, in the hearts subjected to IRI, the significantly increased levels of iNOS and ADMA were observed. The levels were reduced to the level approximate to aerobic control after coadministration of inhibitors. In contrast, NO level (measured as total nitrite/nitrate) was decreased in IRI, compared to aerobic control, and administration of drugs led to its reversion to baseline aerobic level (Figures 3(a)–3(c)).

The results are consistent with existing biological and clinical knowledge. IRI leads to excessive production of ROS [27]. Oxidative stress leads to activation of high-output iNOS and consequently to overproduction of NO which reacts with ROS forming ONOO^−^ and simultaneously limits the bioavailability of NO [28]. Moreover, IRI is associated with increased ADMA level [29]. ADMA is an endogenous competitive inhibitor of NOS. Its accumulation may cause impaired NO synthesis leading to CVD progression [23]. Increased ADMA was shown to be an independent risk factor for coronary artery disease and hypertension and one of the strongest predictors of mortality in patients with myocardial infarction [9]. The increased ADMA level might be a result of oxidative inhibition of dimethylarginine dimethylaminohydrolase (DDAH), which metabolizes ADMA [30] or can be an adaptive mechanism for increased iNOS [25] as we found a positive correlation between ADMA and iNOS (Figure 4(a)).

In I/R, coronary flow was decreased by more than 50% of aerobic control (11.3 vs. 3.34 ml·min^−1^) (Figure 5(b)). There was a negative correlation between ADMA and NO (Figure 4(c)) as well as ADMA and CF (Figure 5(a)). It suggests that decreased CF was a result of increased production of an endogenous inhibitor of NOS-ADMA and thus decreased synthesis of NO by NOS.

It is known that both iNOS and eNOS play a role in cardiac homeostasis [31]. Endothelial NOS is a constitutively expressed regulator of numerous essential cardiovascular functions, mainly responsible for mediation of vasodilation of blood vessels [32]. The activity of the enzyme depends not only on availability of all substrates and cofactors, but also it may be affected by posttranslational modification [31]. The most important of the regulatory eNOS phosphorylation sites is Ser1177, responsible for increasing eNOS activity by 2.5-fold [33]. The phosphorylation occurs in response to many different stimulus, oxidative stress among others [34].

In this study, in the hearts subjected to IRI, we observed the significantly increased levels of eNOS and phospho-eNOS, which were reverted to baseline aerobic levels after coadministration of inhibitors (Figures 6(a) and 6(b)). Overproduction of NO by eNOS is a potential source of ONOO^−^ in the presence of increased iNOS and ADMA, and thus, administration of mixture of drugs leading to decrease in eNOS and phospho-eNOS may serve cardioprotective. Additionally, it was previously shown that eNOS, similarly to iNOS, may change its product profile during I/R from NO to superoxide production [35], and thus, increased expression and activity of eNOS themselves can cause an increase in oxidative stress and the sequelae following oxidative stress such as MMP-2 activation and phosphorylation of MLC. Perkins et al. have shown that attenuating uncoupled eNOS during reperfusion reduced oxidative stress and led to restoration of cardiac function [35]. It is in accordance with our results, where administration of mixture including the NOS inhibitor resulted in normalization of eNOS and phospho-eNOS levels (Figure 6) and improvement of cardiac function at 85% of aerobic control (Figure 2(a)). Taking into account that genetic variations of eNOS/NOS3 may serve as independent risk factors for cardiovascular dysfunction [36], it would be valuable to assess the polymorphism of genes for iNOS, eNOS, and ADMA in the aspect of I/R injury and its treatment by the tested mixture in further studies.

The main reason for heart injury (measured by LDH in coronary effluents, Figure 2(b)) was an increased activity of MMP-2 induced by I/R (Figure 7(a)). It is known that activity of MMP-2 is induced by ONOO^−^ [11], which arises from increased iNOS and ADMA production (uncoupling). Implementation of the inhibitor of matrix metalloproteinases, such as doxycycline, along with inhibitors of NOS (L-NAME) and MLCK (ML-7), even in their subthreshold doses, decreased the activity of MMP-2 and protected heart function (Figure 2(a)).

**Figure 8:**
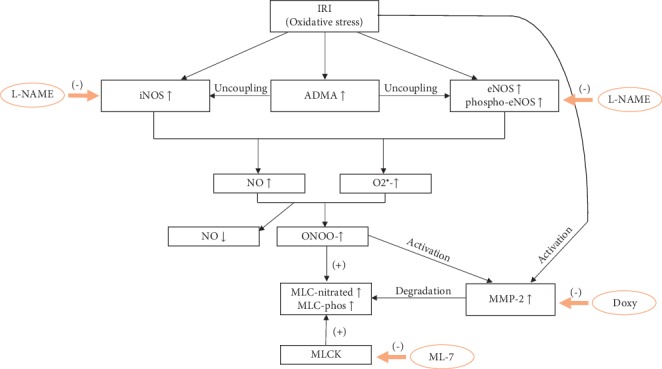
The elements of the cardiac ischemia/reperfusion injury pathway targeted by multidrug therapy with Doxy, L-NAME, and ML-7. Oxidative stress resulting from I/R leads to upregulation of iNOS, eNOS, phospho-eNOS (targeted by L-NAME), and ADMA. ADMA promotes uncoupling of NOS and production of superoxide (O_2_^•−^). Increased level of NOS-derived NO reacts with O_2_^•−^ forming highly reactive peroxynitrite (ONOO^−^), which limits NO bioavailability. ONOO^−^ activates MMP-2 (activity of MMP-2 was targeted by Doxy) which degrades MLC. Increased phosphorylation of MLC by MLCK (targeted by ML-7) increases its susceptibility to MMP-2 degradation. ADMA, asymmetric dimethylarginine; Doxy, doxycycline; eNOS, endothelial nitric oxide synthase; iNOS, inducible nitric oxide synthase; L-NAME, inhibitor of NOS; ML-7, inhibitor of MLC phosphorylation; MLC, myosin light chain; MLC-phos, phosphorylated myosin light chain; MLCK, myosin light chain kinase; MMP-2, matrix metalloproteinase-2; NO, nitric oxide; phopho-eNOS, phosphorylated (S1177) eNOS.

Thanks to the synergistic effect of drugs, the multidrug therapy with the subthreshold doses allows to address a few pathways of I/R injury simultaneously (Figure 8) and to achieve desired result (protection of cardiac function during I/R).

## 5. Conclusion

In conclusion, this study confirmed our previous results [3, 16] that showed that coadministration of subthreshold doses of Doxy, L-NAME, and ML-7 serves cardioprotective. Additionally, this study provided an important insight into understanding the interaction of iNOS, eNOS, and ADMA, which is crucial for the development of the therapy beneficial for patients after myocardial infarction.

## Data Availability

Data supporting the results reported in this article can be found in Wroclaw Medical University, Department of Clinical Chemistry and Laboratory Hematology, Borowska 211A Street, 50-556 Wrocław.
